# Periodontal disease and bronchoaspiration in a neurovegetative patient

**DOI:** 10.5935/0103-507X.20180024

**Published:** 2018

**Authors:** Aline Bergman de Souza Herculano, Kimberly Lampa Gusmão, Diego Silva de Castro, Deisi Carneiro da Costa, Karla Ferreira Dias Saldanha, Ellen Cristina Gaetti-Jardim

**Affiliations:** Residência Multiprofissional, Odontologia, Hospital Universitário, Universidade Federal de Mato Grosso do Sul - Campo Grande (MS), Brasil.; Faculdade de Odontologia, Universidade Federal de Mato Grosso do Sul - Campo Grande (MS), Brasil.; Hospital Universitário, Universidade Federal de Mato Grosso do Sul - Campo Grande (MS), Brasil.; Programa de Pós-Graduação em Ciências da Saúde, Hospital Universitário, Universidade Federal de Mato Grosso do Sul - Campo Grande (MS), Brasil.; Disciplina de Cirurgia Bucomaxilofacial, Universidade Federal de Mato Grosso do Sul - Campo Grande (MS), Brasil.

**To the Editor,**

Teamwork within the hospital setting is fundamental to increase the quality of life of the patient. In this sense, the introduction of hospital dentistry contributes to multidisciplinary and comprehensive health.^(^^1^^)^

Thus, periodontal disease, as an infectious and inflammatory disease, results in destruction of the supporting tissues of the tooth^(^^2^^)^ and is considered a coadjuvant factor or even a precursor of systemic diseases. Aspiration of the teeth, caused by avulsion, can lead to life-threatening accidents, especially in bedridden and unconscious patients. The latency period between the aspiration episode and the onset or worsening of symptoms may be days, months, or years, depending on the degree of obstruction of the airways and the nature of the aspirated foreign body.^(^^3^^)^ It may also occur due to socioeconomic, cultural, and even population custom factors,^(^^4^^)^ which is consistent with the possibility of dental aspiration by habits of deficient hygiene, especially in hospitalized patients, in addition to the possibility of aspiration pneumonia, especially in elderly patients.

A 56-year-old male patient was seen in the dentistry department at a hospital in Campo Grande (MS) with cerebral hypoxia post-cardiorespiratory arrest. Despite normal-colored mucosa and preserved salivary flow, a precarious oral condition was observed during the clinical examination: traumatic injury to the lower lip, secretion adhered to the dental surface, partial edentulism, cervical caries, supra and subgingival calculus, and varying degrees of dental mobility, characterizing periodontal disease (Figure 1A). Radiographic examination revealed the presence of two foreign bodies in the right lung, similar to two dental elements (Figure 1B).

**Figure 1:**
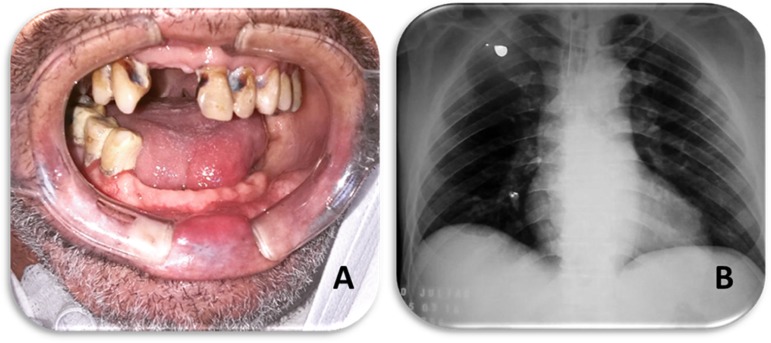
Clinical aspect and consequence of advanced periodontal disease. (A) Front view. Initial clinical evaluation showing a precarious oral condition, with a large accumulation of dental calculi and caries. (B) Posteroanterior radiograph of the thorax showing two dental elements in the right lung.

Because the patient was tracheostomized and under the abovementioned conditions, the indicated treatment plan was total exodontia due to the evident possibility of new bronchoaspiration. However, the uncertain treatment history led to the option for bedside care for the removal of infectious foci, divided into sessions, with satisfactory results. Bronchoaspiration of dental elements may result in pulmonary irritation, marked microbial proliferation, pneumonia, and death of the patient.

Interdisciplinary interactions contribute to a reduction in the hospitalization duration and avoid disastrous consequences in the hospital setting. Thus, the dental surgeon plays a fundamental role in improving patient quality of life since the oral condition changes the evolution and response to medical treatment. The patient described herein is under palliative and dental care.

The patient under palliative care deserves and should receive dental care to control possible painful symptoms and severe odontogenic infections, mainly due to the incapacity for self-care. The literature presents the need for frequent dental brushing and care with possible prosthetic devices to minimize the acute risk of aspiration of this oral microbiota, favoring or aggravating the patient's systemic condition, especially in relation to pneumonia.^(^^5^^)^

*Aline Bergman de Souza Herculano* *Multiprofessional Residency, Dentistry, Hospital Universitário,
Universidade Federal de Mato Grosso do Sul - Campo Grande (MS), Brazil.* *Kimberly Lampa Gusmão* *Faculdade de Odontologia, Universidade Federal de Mato Grosso do Sul - Campo
Grande (MS), Brazil.* *Diego Silva de Castro* *Multiprofessional Residency, Dentistry, Hospital Universitário,
Universidade Federal de Mato Grosso do Sul - Campo Grande (MS), Brazil.* *Deisi Carneiro da Costa* *Hospital Universitário, Universidade Federal de Mato Grosso do Sul -
Campo Grande (MS), Brazil.* *Karla Ferreira Dias Saldanha* *Postgraduate Program in Health Sciences, Hospital Universitário,
Universidade Federal de Mato Grosso do Sul - Campo Grande (MS), Brazil.* *Ellen Cristina Gaetti-Jardim* *Discipline of Bucomaxilofacial Surgery Universidade Federal de Mato Grosso
do Sul - Campo Grande (MS), Brazil.*

## References

[1] Godoi AP, Francesco AR, Duarte A, Kemp AP, Silva-Lovato CH (2009). Hospital odontology in Brazil. A general vision. Rev Odontol UNESP.

[2] Darveau RP, Tanner A, Page RC (2000). The microbial challenge in periodontitis. Periodontol.

[3] Gonçalves ME, Cardoso SR, Rodrigues AJ (2011). Foreign body in the airway. Pulmão RJ.

[4] Karapolat S (2008). Foreign-body aspiration in an adult. Can J Surg.

[5] van Der Maarel-Wierink CD, Vanobbergen JN, Bronkhorst EM, Schols JM, de Baat C (2013). Oral health care and aspiration pneumonia in frail older people:
a systematic literature review. Gerodontology.

